# Clinical Outcomes of Transplanted Kidneys from Deceased Donors Using Different Generic Preservation Solutions

**DOI:** 10.3390/medicina58111579

**Published:** 2022-11-02

**Authors:** Aureliusz Kolonko, Natalia Słabiak-Błaż, Robert Król, Andrzej Więcek

**Affiliations:** 1 Department of Nephrology, Transplantation and Internal Medicine, Medical University of Silesia, 40-027 Katowice, Poland; 2 Department of General, Vascular and Transplant Surgery, Medical University of Silesia, 40-027 Katowice, Poland

**Keywords:** cold organ storage, kidney transplantation, immediate graft function, graft survival, preserving solution

## Abstract

*Background and Objectives*: StoreProtect Plus^®^ is a preserving solution for cold organ storage, with a composition identical to Institute Georges Lopez (IGL-1) solution. The aim of this single center study was to compare the clinical performance of StoreProtect Plus with the generic counterpart of University of Wisconsin preservation fluid, named SPS-1^®^. *Materials and Methods*: The clinical outcomes of 168 consecutive organs preserved with StoreProtect Plus solution and 167 organs preserved with SPS-1 solution were compared. During an 18-month post-transplant follow-up period, kidney graft function, the frequency of acute rejection, post-transplant diabetes, and infectious complications, as well as patient and graft survival were analyzed. *Results:* There was significantly more immediate graft function (IGF) (39.3 vs. 24.0%; *p* < 0.01) and less slow graft function (SGF) (38.7 vs. 51.5%; *p* < 0.05) in the StoreProtect Plus group in comparison with the SPS-1 group, whereas the occurrence of DGF was similar in both groups. Long-term kidney graft function was comparable. Multivariate regression analysis showed that the use of StoreProtect Plus vs. SPS-1 solution (r_partial_ = 0.217; *p* < 0.001) and the amount of residual diuresis (r_partial_ = 0.147; *p* < 0.001) independently increased the occurrence of IGF, whereas Scr > 1.5 mg/dL prior to organ procurement (r_partial_ = −0.198; *p* < 0.001), longer CIT (r_partial_ = −0.170; *p* < 0.01), and CVD donor death (r_partial_ = −0.214; *p* < 0.001) were associated with SGF. *Conclusions:* The higher occurrence of IGF was found in kidney transplant recipients whose organs were preserved using StoreProtect Plus solution as compared with SPS-1 solution. The two groups did not differ in kidney graft function, the frequency of post-transplant complications, as well as patient and graft survival.

## 1. Introduction

In kidney transplantation, organ preservation solutions have been developed to reduce ischemia/reperfusion tissue injury during cold storage and, in consequence, to improve early graft function and its long-term survival [1]. The aim of hypothermia is to decrease the metabolic rate during anaerobic conditions by limiting adenosine triphosphate consumption and inhibiting cellular enzymatic activity, moderating the cellular degradation by phospholipid hydrolysis [1,2]. University of Wisconsin (UW) solution is the current standard and most often used preservation solution in solid organ transplantation. However, over past decades, several other preservation fluids have been introduced in clinical practice in order to optimize the biologic and metabolic conditions during pretransplant organ maintenance, including histidine-tryptophan-ketoglutarate (HTK) and Institute Georges Lopez-1 (IGL-1) solutions. Each solution varies in composition; however, the key strategy is the use of colloids, impermeants, electrolytes, antioxidants, and nutrients, to minimize ischemic/hypoxic injury and to improve kidney graft function after reperfusion [3,4]. It is crucial especially in the case of organs procured from extended criteria donors (ECD), whose percentage has been steadily growing in the last decades [5]. 

In recent years, the use of generic preservation solutions has been increasing as a result of a worldwide transplant cost-saving strategy. However, although the fluid composition of a generic product should be the same as in the original solution, other factors, including the purity of the components used, the performance/quality of the bags, and storage conditions, could affect the graft viability and, in consequence, worsen the clinical outcomes of organ transplantation [6]. It is worth noting that some solutions contain labile molecules, such as glutathione or polyethylene glycols, which are very sensitive to the storage conditions and may be more prone to degrade at room temperature [6]. This applies, among others, both to UW and IGL-1 solutions. Therefore, the concerns about the functional similarity and bioequivalence of generic preservation solutions are fully justified and should be investigated by clinical studies. Unfortunately, except for the above cited in vitro study comparing the original Celsior solution and its European generic counterpart, we did not find any other comparative study in this field. Whereas comparative studies between the original UW and IGL-1 solutions are available both in kidney [7,8] and liver [9] transplantation settings, there are no direct comparisons of their generic products.

StoreProtect Plus^®^ is a solution for the perfusion and cold storage of organs for transplantation, produced by Carnamedica (Carnamedica, Warsaw, Poland) and marketed since 2017 (ID: 9619 7077 2240). Its composition is identical to IGL-1 solution. It meets the requirements of the European Medical Devices Directive (MDD) and is certified by the Notifying Authority No. 2274. From December 2018, it has been constantly used in our center for organ preservation during kidney, liver, and simultaneous pancreas–kidney transplantation. Taking into account that the original UW solution (Viaspan, Bristol-Meyers Squibb GmbH, Munich, Germany) was used in our center over a decade ago, with different characteristics of both recipients and donors, as well as markedly different immunosuppression and induction therapy protocols, we decided to perform a retrospective comparison of the generic UW solution (SPS-1, Organ Recovery System, Diegem, Belgium) with the generic IGL-1 solution (StoreProtect Plus) in aspects of early post-transplant graft function and also midterm follow-up patient and graft clinical outcomes.

## 2. Materials and Methods

### 2.1. Study Groups

In the period from December 2018 to December 2020, 168 consecutive organs were transplanted and preserved with StoreProtect Plus preservation fluid. The control group consisted of 167 consecutive kidney transplantations between May 2015 and November 2016, with organs preserved with SPS-1 solution. Due to the potential risk of bias caused by the relatively short cold preservation time, the recipients of organs from living donors (*n* = 7) and simultaneous pancreas–kidney transplant patients (*n* = 10) were not included in the analysis. All organs were preserved in cold storage and all donors and recipients were Caucasian.

The analysis was performed using a center-operated prospective transplant database, containing clinical data of donors and recipients. Donor data included age, body mass index (BMI), ECD status, the Kidney Donor Risk Index (KDRI) value, the occurrence of hypertension, the serum creatinine level prior to organ procurement, and the cause of death. There were no diabetic donors in the analyzed cohort. 

The study protocol was reviewed by the Bioethical Committee of the Medical University of Silesia (PCN/CBN/0052/KB/128/22). 

### 2.2. Kidney Graft Function

Initial graft function was defined based on the serum creatinine (Scr) level at post-transplant day 3 and the need for dialysis therapy during the first week after transplantation. Patients with immediate graft function (IGF) were characterized by Scr < 3 mg/dL at day 3; slow graft function (SGF) was defined as Scr above 3 mg/dL at day 3, and delayed graft function (DGF) was recognized in patients who required dialysis therapy after transplantation. The latter category was also used in cases of ineffective post-transplant diuresis (i.e., polyuria without substantial Scr lowering) during the first postoperative days, which however, usually enables those patients not to be dialyzed due to the lack of overhydration and hyperkalemia. Primary graft nonfunction (PGN) was attributed to those kidney grafts which had to be removed during the first post-transplant hospitalization.

Scr was analyzed at postoperative (POD) day 3 and 7, and at the day of discharge from the hospital. In patients with DGF, the morning predialysis Scr values were analyzed. In the follow-up period, Scr was analyzed 3-, 6-, 12-, and 18-months post-transplantation. The estimated glomerular filtration rate (eGFR) was calculated using the Modification of Diet in Renal Disease (MDRD) formula.

### 2.3. Patient and Graft Outcomes

During the 18-month follow-up period, patient and graft survival were analyzed. Additionally, we analyzed the frequency of acute rejection (AR) episodes, post-transplant diabetes mellitus (PTDM), and infectious complications, including CMV infection.

### 2.4. Statistical Analysis

Statistical analyses were performed using STATISTICA 13.3 (Tibco Inc., Palo Alto, CA, USA) and MedCalc 18.6 (MedCalc Software, Ostend, Belgium). Data were presented as means with a 95% confidence interval (CI), medians with Q1–Q3 values, or frequencies. For the comparison of study groups, the Student *t*-test (for quantitative variables) or the χ^2^ test (for qualitative variables) was used. Variables with nonparametric distribution were compared using the Mann–Whitney U test. Correlations were calculated using Spearman. Stepwise multivariate regression analysis was performed for IGF as the dependent variable, including dialysis vintage, residual diuresis, cold ischemia time (CIT), the use of induction therapy, the type of preservation solution used (StoreProtect Plus vs. SPS-1 solution), donor BMI, hypertension, and last donor Scr prior to organ procurement > 1.5 mg/dL as potential independent variables. The model included all variables selected in the univariate analysis, CIT as a traditional well-established risk factor, and the type of preservation solution used. In all the statistical tests, ‘*p*’ values below 0.05 were considered statistically significant. 

## 3. Results

### 3.1. Study Groups

The final analysis included 168 KTRs who received organs preserved with StoreProtect Plus and 167 KTRs who received organs preserved with UW. The 18-month results were available for the entire study cohort. In the whole study group (*n* = 335), the causes of chronic renal disease were as follows: chronic glomerulonephritis (38.8%), interstitial nephritis (7.5%), polycystic kidney disease (16.1%), diabetic nephropathy (8.1%), hypertensive nephropathy (11.6%), and other disease and unknown conditions (17.9%). 

During the entire study period, the initial immunosuppressive regimen was based on tacrolimus, mycophenolate mofetil, and steroids. Additionally, 80% of the patients received an induction therapy, using monoclonal (basiliximab, IL-2 RA) or polyclonal (antithymocyte globulin, ATG) antibodies. There was a higher percentage of patients with induction therapy in the StoreProtect Plus group as compared with the SPS-1 group (99.4 vs. 60.5; χ^2^ = 80.4, *p* < 0.001). Both analyzed groups did not differ in regard to age, sex, BMI, primary kidney disease, HLA mismatch, CIT, and the percentage of repeated transplantations (Table 1). The SPS-1 group was characterized by a significantly longer time of pretransplant dialysis therapy, lower residual diuresis, and a greater percentage of patients with last PRA > 25%. On the other hand, the donors from the StoreProtect Plus group had higher BMI and were more frequently hypertensive. Moreover, there were also more cerebrovascular and cardiovascular deaths in this group in comparison with the SPS-1 group (χ^2^ = 6.3, *p* < 0.05) (Table 1).

### 3.2. Early Kidney Graft Function

No significant difference was found in the occurrence of DGF between the two study groups. Importantly, there was significantly more IGF and less SGF in the StoreProtect Plus group than in the SPS-1 group (Table 2). The frequency of PGN did not differ between the groups. As expected, the occurrence of IGF was more than twice as frequent in patients transplanted with organs procured from a standard criteria donor (SCD) as compared with an ECD (SPS-1 group: 28.7 vs. 11.1%; *p* < 0.05; StoreProtect Plus group: 47.3 vs. 23.2%; *p* < 0.01). Interestingly, the percentage of SGF was similar after SCD and ECD transplantations, whereas the significant impact of ECD status on DGF occurrence was shown (SPS-1 group: 35.6 vs. 18.9%; *p* < 0.01; StoreProtect Plus group: 30.4 vs. 14.3%, respectively, *p* < 0.05).

Additionally, in the StoreProtect Plus group, there were significantly lower median serum creatinine levels measured at the 3rd, 7th, and discharge day as compared with the SPS-1 group (Table 2). Of note, the median length of the first post-transplant hospital stay was similar (14 vs. 15 days; *p* = 0.60). As expected, donor Scr was associated with recipient Scr during the hospital stay (POD 3: R = 0.240; POD 7: R = 0.240; day of discharge: R = 0.177; all *p* < 0.001). 

Stepwise multivariate regression analysis revealed that the use of StoreProtect Plus vs. SPS-1 solution (r_partial_ = 0.217; *p* < 0.001) and residual diuresis (r_partial_ = 0.147; *p* < 0.01) independently increased the occurrence of IGF, whereas Scr > 1.5 mg/dL prior to organ procurement (r_partial_ = −0.198; *p* < 0.001), longer CIT (r_partial_ = −0.170; *p* < 0.01), and CVD donor death (r_partial_ = −0.214; *p* < 0.001) were associated with slower graft function. The rest of the potential explanatory variables were removed from the final model.

### 3.3. Patient and Graft Outcomes during the Follow-Up Period

The 18-month follow-up data were available for all the patients in the SPS-1 group and for 164 (97.6%) patients in the StoreProtect Plus group. During this period, there were six graft losses (3.6%) in each group. Fourteen patients (8.4%) died in the SPS-1 group and 10 patients (6.0%) died in the StoreProtect Plus group (*p* = 0.39). The structure of death causes was comparable, with 50% infectious death in each group and 21.5 vs. 30% CVD death, respectively (*p* = 0.64). 

The incidence of AR during the follow-up period was similar in both groups (23 vs. 22 events; *p* = 0.84). In addition, the percentage of patients with PTDM was comparable (14.9 vs. 16.2%; *p* = 0.75). Finally, the rate of all infectious complications during the first 18 months post-transplantation, including CMV episodes, did not differ between groups (35 vs. 36%; *p* = 0.85). 

Kidney graft function did not differ between the groups up to the 18-month timepoint (Table 2). In the whole study group, there was a positive correlation between donor Scr and kidney graft function up to the 18-month timepoint.

## 4. Discussion

In the present analysis, it was found that the clinical performance of the generic preservation solution, StoreProtect Plus, is not inferior to the previously used generic SPS-1 solution, when taking into account both early post-transplant kidney graft function and the 18-month follow-up observation. There was a substantially higher occurrence of IGF in the StoreProtect Plus group. Importantly, the 12-month kidney graft function in both the analyzed groups was satisfactory and comparable to the previously reported data from the modern immunosuppression era [10,11]. 

Our current results regarding the clinical effectiveness of two different generic organ preservation solutions are similar to the previous reports, comparing the efficacy of their original counterparts [7,8] in kidney transplantation. The first of those studies focused on inflammation, reactive oxygen species production, proximal tubule damage, proteinuria, histology, and renal function, whereas the second study analyzed the occurrence and duration of DGF, as well as eGFR, up to 1-year post-transplant. Interestingly, the adjusted risk for DGF was significantly lower for IGL-1 as compared to any other solution, including UW [7], which is in line with the more frequent IGF occurrence in the IGL-1 counterpart solution in our study. 

It is worth noting that several different SGF definitions were used in previous studies, being generally based on the Scr level measured at POD 5, 7, or even 14, or on the inadequate Scr reduction rate over a given period [12]. Therefore, proper interpretation and comparison of the study results is very troublesome. In several studies using SGF criteria based on Scr > 3 mg/dL at POD 5, the 3-year [13] and 5-year [14,15] graft survival was lower in the SGF group as compared with IGF. Importantly, the occurrence of AR episodes was also higher in patients with postoperative SGF [13,16]. Additionally, the significantly worse kidney graft survival among SGF than IGF patients was noted only in the presence of AR [16]. It suggests that the concomitant rejection process may be one of the underlying SGF causes, which might be an argument for performing the protocol biopsy in each kidney transplant recipient without IGF to identify those with subclinical rejection. 

In contrast to the abovementioned most common SGF definition, our transplant center adopted the stricter criterion of Scr > 3 mg/dL at POD 3, which resulted in the relatively low IGF percentage reported in the present study compared to others [13,14,15,16]. Notably, patients in the StoreProtect Plus group were characterized by a similar frequency of IGF to patients in the study by Nel et al. [17]. They used an SGF definition based on Scr > 1.7 mg/dL at POD 5, which is clinically comparable with our study, despite the markedly older donor and recipient age in our present cohort. In contrast, the IGF occurrence in the SPS-1 group was significantly lower. 

The main limitation of this study is its retrospective character. However, due to the internal regulations, the type of preservation solution used during the given time period was chosen independently by our hospital supply department, and so it is difficult to plan prospective pair-to-pair comparison. Nevertheless, it is the first clinical study to analyze and compare the results of kidney transplantations using two generic preservation fluids, based on the UW and IGL-1 original solutions. Another limitation is the fact that both investigated groups of KTRs were not transplanted contemporarily.

## 5. Conclusions

To conclude, the higher occurrence of IGF was found in kidney transplant patients whose organs were preserved using StoreProtect Plus solution as compared with SPS-1 solution, whereas the occurrence of DGF was similar. However, the two analyzed groups did not differ in kidney graft function, the frequency of post-transplant complications, as well as patient and graft survival during the 18-month follow-up period.

## Figures and Tables

**Table 1:** Clinical characteristics of the study groups.

	SPS-1 N = 167	StoreProtect Plus N = 168	*p*
**Recipient and transplant characteristics**			
Age (years)	50.0 (48.1–51.8)	49.6 (47.7–51.5)	0.80
Sex (M/F)	90/77	103/65	0.17
BMI (kg/m^2^)	25.3 (24.7–25.9)	25.6 (25.0–26.3)	0.39
Dialysis vintage (months) *	35 (22–62)	30 (18–45)	<0.05
Residual diuresis (mL) *	300 (0–1000)	500 (50–1500)	<0.05
Hypertension (*n* (%))	151 (90.4)	158 (94.0)	0.62
Diabetes (*n* (%))	18 (10.8)	21 (12.5)	0.62
ESRD cause (*n*) Glomerulonephritis Diabetes Pyelonephritis ADPKD Hypertensive nephropathy Other Unknown	63 14 14 21 26 10 19	67 13 11 33 13 7 24	0.20
HLA class I mismatch	2.3 (2.1–2.4)	2.3 (2.2–2.4)	0.65
HLA class II mismatch	0.61 (0.52–0.70)	0.58 (0.50–0.65)	0.57
PRA max (%) *	0 (0–10)	0 (0–3)	0.11
Retransplantation (*n* (%))	34 (20.4)	25 (14.9)	0.19
CIT (h)	18.0 (17.0–18.9)	17.3 (16.4–18.2)	0.32
CIT > 18 h (*n* (%))	79 (47.3)	79 (47.0)	0.88
Induction therapy (*n* (%))	101 (60.5)	167 (99.4)	<0.001
Induction structure No induction IL-2RA (%) ATG (%)	66 55 46	1 102 65	<0.001
**Donor characteristics**			
Age (years)	46.6 (44.6–48.5)	45.4 (43.5–47.3)	0.39
Sex (M/F)	99/68	110/58	0.24
BMI (kg/m^2^)	25.0 (24.5–25.5)	26.4 (25.8–26.9)	<0.001
SBP *† (mmHg)	127 (120–140)	130 (120–140)	0.85
DBP *† (mmHg)	78 (70–85)	80 (70–80)	0.62
ECD status (*n* (%))	45 (27.0)	56 (33.3)	0.22
S_cr_ > 1.5 mg/dL *† (*n* (%))	37 (22.2)	58 (34.5)	0.02
Hypertension (*n* (%))	34 (20.4)	52 (31.0)	<0.05
Cardiac arrest (*n* (%))	28 (16.8)	36 (21.4)	0.28
Cause of death (*n*) CVD Trauma Other	86 61 20	109 46 13	<0.05
KDRI *	1.164 (0.930–1.434)	1.217 (0.935–1.472)	0.72

Data presented as means with 95% confidence interval, except * medians with Q1–Q3 values. † prior to the organ procurement. BMI, body mass index; ESRD, end-stage renal disease; ADPKD, autosomal dominant polycystic kidney disease; PRA, panel reactive antibodies; HLA, human leukocyte antigen; CIT, cold ischemia time; IL2-RA, interleukin 2 receptor antagonist; ATG, antithymocyte globulin; SBP, systolic blood pressure; DBP, diastolic blood pressure; ECD, extended criteria donor; Scr, serum creatinine; CVD, cardiovascular death; KDRI, kidney donor risk index.

**Table 2:** The kidney graft function in patients in the study groups, based on the type of cold storage solution.

	SPS-1	StoreProtect Plus	*p*
Early kidney graft function			
IGF (*n* (%))	40 (24.0)	66 (39.3)	<0.01
SGF (*n* (%))	86 (51.5)	65 (38.7)	<0.05
DGF (*n* (%))	39 (23.4)	33 (19.6)	0.41
PGN (*n* (%))	2 (1.2)	4 (2.4)	0.41
DGF duration (days)	6 (3–9)	8 (4–11)	0.41
Serum creatinine level (mg/dL)			
3rd POD	6.1 (3.1–9.3) N = 164	4.1 (2.2–7.3) N = 164	<0.001
7th POD	3.0 (1.5–6.3) N = 165	1.8 (1.1–5.0) N = 163	<0.001
Discharge day	1.6 (1.2–2.2) N = 159	1.3 (1.1–1.9) N = 162	<0.001
3-month	1.3 (1.1–1.5) N = 154	1.3 (1.0–1.5) N = 160	0.89
6-month	1.2 (1.0–1.5) N = 153	1.3 (1.1–1.5) N = 156	0.48
12-month	1.2 (1.0–1.5) N = 147	1.2 (1.0–1.5) N = 155	0.22
12-month eGFR (mL/min/1.73 m^2^)	58.8 (46.2–73.3)	59.0 (48.9–69.4)	0.74
18-month	1.2 (1.0–1.5) N = 147	1.3 (1.0–1.5) N = 152	0.19

Data presented as medians with interquartile range or frequencies. Statistics: Mann–Whitney U test or χ^2^ test. IGF, immediate graft function; SGF, slow graft function; DGF, delayed graft function; PGN, primary graft nonfunction; POD, postoperative day; eGFR, estimated glomerular filtration rate.

## Data Availability

The data presented in this study are available on reasonable request from the corresponding author.
